# Advances in Functional Genomics for Human Health

**DOI:** 10.3390/genes17070763

**Published:** 2026-06-30

**Authors:** Patrick R. Gonzales

**Affiliations:** Department of Pathology and Laboratory Medicine, University of Kansas Medical Center, Kansas City, KS 66160, USA; pgonzales@kumc.edu; Tel.: +1-(913)-574-0288

**Keywords:** functional genomics, structural variation, copy number variation, CRISPR-Cas, cytogenomics, topologically associating domain (TAD)

## Abstract

Cytogenomics, including karyotyping, FISH, chromosomal microarrays, and optical genome mapping, has yielded significant results for clinical phenotypes in constitutional and cancer genetics, including intellectual disability, autism spectrum disorders, dysmorphic features, and hematological and solid-tissue neoplasia. However, some of these assays have yielded results of unclear significance because the abnormalities detected were often located in intergenic regions of the genome. Because these abnormalities are within the “dark matter” of the genome, their clinical significance has been a matter of speculation. However, functional genomics can explore the clinical implications of such abnormalities more robustly, whether the abnormalities disrupt topologically associating domains (TADs), delete regulatory regions, etc. Some human genetic diseases associated with these intergenic abnormalities and characterized by functional genomics include preaxial polydactyly (*SHH* gene), Pierre Robin syndrome (*SOX9*), and 5q14.3 microdeletion syndrome (*MEF2C*). While functional genomics is a broad research topic, this review focuses on prior and current efforts to leverage functional genomics within the intergenic regions for human health.

## 1. Introduction

The human genome is not a single static entity but is collectively shared among all humanity with a multitude of unique and recurrent structural and copy-number variants. While the reference genome is a representation of a few well-characterized humans, several seminal studies noted the detection of large-scale structural variants (SVs) and submicroscopic copy number variants (CNVs) that diverged from this reference genome [1,2,3]. Some of these structural and copy number variants were found to play a role in human disease via “dosage effects” through gain or loss of genes important in developmental or regulatory pathways, or through “position effects” via perturbation of proper gene expression as a consequence of structural variation displacing regulatory machinery [4]. 

Noteworthy among these SVs and CNVs was the discovery that many involved regions of the genome that do not code for proteins, encompassing approximately 98% of the genome and including what has been colloquially called the “dark matter” of the genome [5]. Some human genetic diseases associated with these intergenic abnormalities include enhanceropathies [6], or pathogenic alterations of enhancer function, such as preaxial polydactyly (Involving regulation of the *SHH* gene) [7], Pierre Robin syndrome (*SOX9*) [8], and 5q14.3 microdeletion syndrome (*MEF2C*) [9].

Groundbreaking work to characterize the cause and effects of these variants has spawned the field of functional genomics. The Encyclopedia of DNA Elements (ENCODE) project consortium systematically mapped functional elements encoded in the human genome, including regions of transcription, transcription factor association, chromatin structure, and histone modifications, enabling assignment of biochemical function to over 80% of the genome [10,11]. The Genotype-Tissue Expression (GTEx) project consortium have focused on structural variation as contributors to human genetic disease across multiple human tissue types [12,13]. Functional genomics includes techniques that assess effects of expression quantitative trait loci (eQTLs) as well as genetic effects on the three-dimensional chromatin state [14]. Functional genomics has emerged as a tool in human cytogenomics through its ability to characterize the effects of putative copy number and structural disruptions in intragenic and intergenic space to generate abnormal human phenotypes, in effect becoming functional cytogenomics [15]. This review summarizes some of the most noteworthy studies that track the development of functional genomics and also highlights some recent studies that show the benefits of functional genomics as a complement to traditional molecular cytogenomic techniques, to fully characterize cytogenomic abnormalities and to advance human health.

## 2. Seminal Studies and Techniques

Although the overwhelming majority (~98%) of the human genome has been shown not to code for proteins, work by Bejerano and colleagues showed that there were sequences in the human genome of 200 bp or greater in length that were shared at 100% identity between humans, mice, and rats, nearly all of which were non-coding, and were therefore called ultraconserved elements (UCEs) [16]. Bejerano’s group hypothesized that these 481 sequences were constrained from sequence divergence over several million years of evolutionary time because they served as essential functional elements in genome structure or regulation in mammals and other vertebrates. Additional work by Stephens and colleagues found 2189 UCEs ≥200 bp to be found in at least three of five placental mammals, and are associated with genes that are involved in the regulation of transcription and development, further confirming the necessity of these sequences for mammalian genome function [17].

Pennacchio and colleagues tested the putative functions of these UCEs, as well as other genomic sequences conserved between humans and pufferfish, as enhancer elements of gene regulation using transgenic heat-shock promoter/*lacZ* reporter constructs in embryonic mice at 11.5 days (e11.5) [18]. The mice at this embryonic stage were amenable to whole-mount staining and whole-embryo visualization, including many of the major tissues and organs, and the assays were designed with the expectation that ultraconserved elements tend to be enriched and clustered in genes active during embryonic development. The majority of these enhancers drove expression in only one anatomical structure in the embryos, with the highest localization being in the forebrain, followed by the midbrain, neural tube, hindbrain, limb, eye, and dorsal root ganglia. Their work showed that non-coding ultraconserved genomic elements are involved in tissue-specific gene expression.

While prior groups examined the function of small sequences in the human genome, Lieberman-Aiden and colleagues investigated three-dimensional (3D) long-range interactions in the genome using Hi-C, involving chromosome/chromatin conformation capture via crosslinking of genomic DNA, digestion, ligation, shearing and paired-end next-generation sequencing (NGS) [19]. Their work confirmed the presence of distinct chromosome “territories” in the nucleus, co-localization of smaller gene-rich chromosomes, and spatial segregation of open and closed chromatin compartments consistent with early- and late-replicating chromatin, respectively, denoted as “A” and “B” compartments, at a resolution of ~1 Mb. They also showed that the chromatin was packed in an orderly fashion within the nucleus as a “fractal globule,” allowing for easily accessible and regulatable genomic packaging.

Work by Dixon and colleagues further explored these long-range interactions in humans and mice with embryonic and terminally differentiated cells using Hi-C, where they described large, megabase-sized local chromatin interaction domains, which they called “topological domains”, later to be called topologically associating domains (TADs) [20]. Using chromatin immunoprecipitation sequencing (ChIP-Seq) data, these TADs were shown to be bound on their flanks by enrichment of CTCF-binding sites, also known as insulators, enrichment of H3K9me3 epigenetic marks consistent with heterochromatin in differentiated cells, and co-localization with housekeeping genes. The TAD boundaries also appeared to be invariant across cell types. Interactions between loci within the TADs were much higher than between loci across TAD boundaries, which is consistent with the proximity of genes to cis-regulatory elements, including enhancers. The TADs were also shown to maintain their overall structure between humans and mice, as shown by conservation across syntenic blocks of genomic material between the two species. The group closely examined the *Hoxa* gene cluster in mice as an example of TADs playing a role in gene regulation, with a CTCF insulator coinciding with a TAD boundary between two spatially close *Hoxa* gene groups. Also noteworthy was the retention of a highly similar TAD structure in the syntenic *HOXA* cluster in humans.

**Figure 1:**
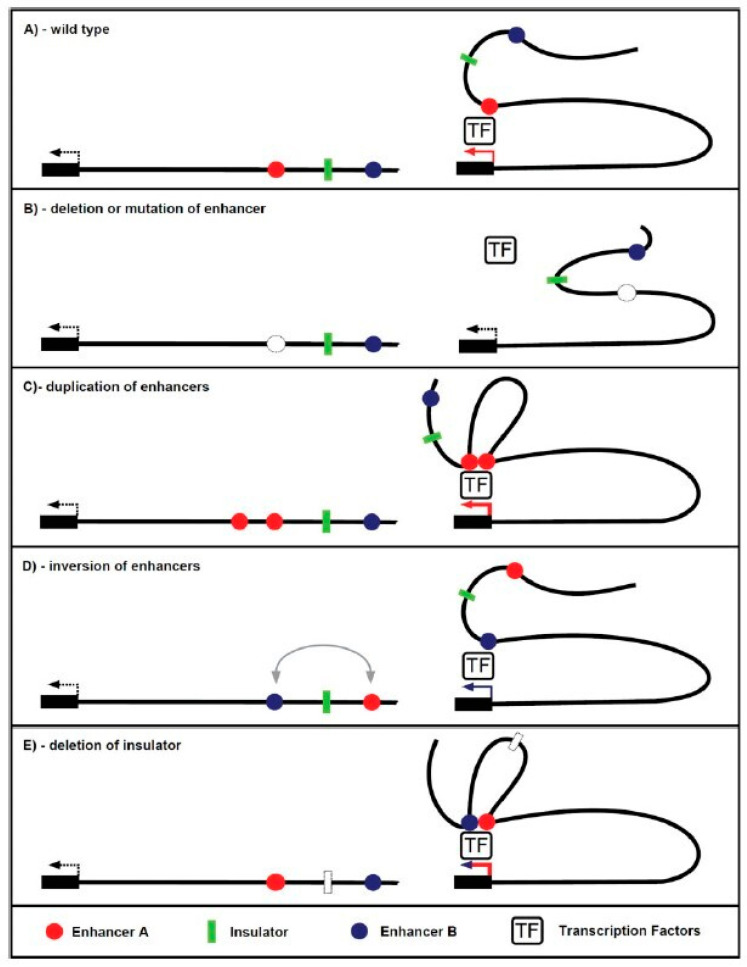
Cis-regulatory variants alter the regulation of developmental genes. (**A**) Red enhancer interacts with transcription factor (TF) to drive expression of target gene; (**B**) deletion of red enhancer abolishes interaction and expression; (**C**) duplication of red enhancer results in overexpression; (**D**) Inversion of enhancers results in ectopic expression of target gene with non-native enhancer; (**E**) deletion of insulator results in mis-expression (Adapted from [21] with permission).

**Figure 2:**
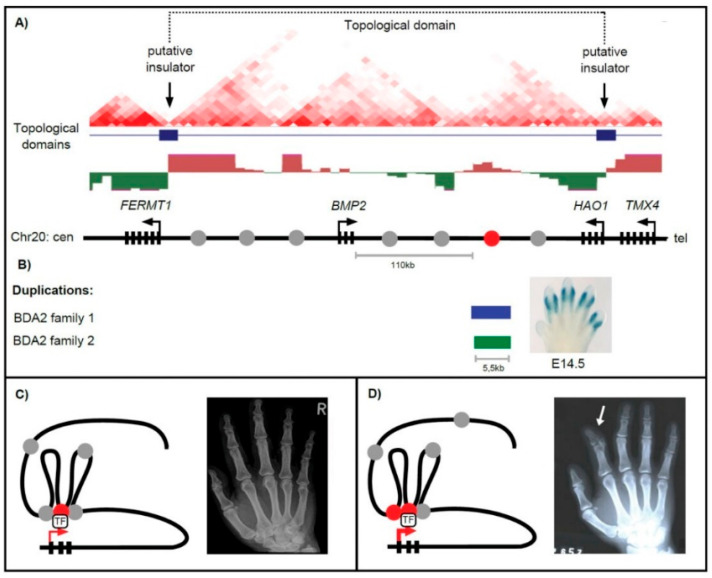
Duplications in the regulatory landscape of *BMP2* with BDA2. (**A**) TAD of *BMP2* with red enhancer duplicated in BDA2 families; (**B**) duplication regions of BDA2 families with *lacZ* assay showing enhancer activity in e14.5 distal limb buds of mouse; (**C**) wild-type expression model for *BMP2*; (**D**) expression model for duplicated *BMP2* enhancer (Adapted from [21] with permission).

Spielmann and Mundlos reviewed some of the mechanisms by which structural variation could disturb proper gene expression by disrupting the cis-regulatory landscape [21]. They showed how deletions, duplications, and inversions of enhancers, and the deletion of insulators can alter gene expression and generate an abnormal phenotype (Figure 1). Datasets from two families with brachydactyly type A2 (BDA2) showed two similarly located duplications within a TAD containing the morphogenetic protein gene *BMP2*. These duplications were ~110 kb downstream of *BMP2* and contained a conserved likely enhancer sequence of ~660 bp, with the duplication perturbing the proper expression of BMP2 protein in the developing limb bud and resulting in the brachydactyly phenotype (Figure 2).

Evolutionary stability of TADs was studied by McArthur and Capra to assess whether complex-trait heritability and sequence conservation were stable within TADs versus at TAD boundaries [22]. They also tested whether stable TAD boundaries, or boundaries invariant across multiple cell types, play a role in complex trait heritability and sequence conservation. Using statistical modeling of GWAS studies and TAD maps against 37 different cell types, they found that TAD boundaries are indeed enriched for complex-trait heritability for 41 traits and are evolutionarily constrained at the sequence level across all cell lines tested, as opposed to within TADs. They also found that some TAD boundaries are unique to specific cell lines, but also that cell lines of similar lineages shared many of these variable TAD boundaries, and thus provide preliminary evidence that there is cell-type specificity in the 3D genome. 

**Figure 3:**
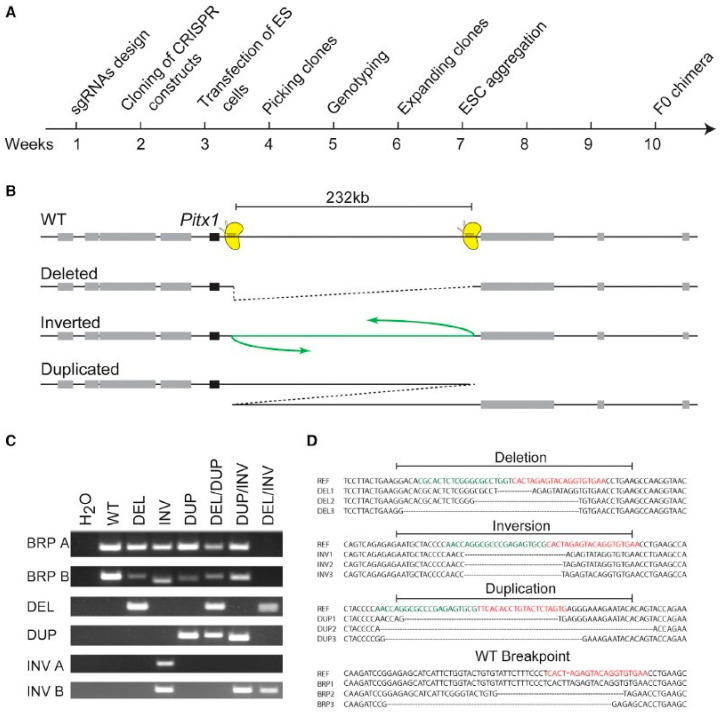
CRISPR/Cas (CRISVar) to generate structural and copy number variants in mice. (**A**) Overview and timeline of CRISVar; (**B**) variants generated in the *Pitx1* locus by CRISPR; (**C**) PCR confirmation of CRISPR constructs at breakpoints; (**D**) sequence variability at breakpoints using CRISPR (Adapted from [23] with Open Access).

**Figure 4:**
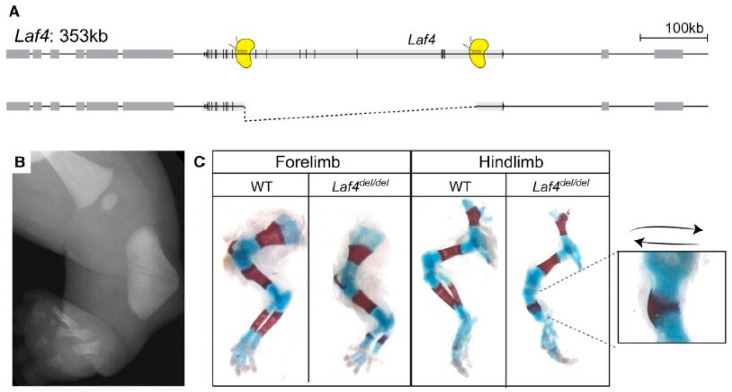
350 kb deletion at *Laf4* recapitulates Nievergelt syndrome. (**A**) *Laf4* genomic region, with CRISPR/Cas binding sites in yellow, deletion shown as dashed line; (**B**) radiograph of patient with lower limb abnormalities; (**C**) skeletal preparations of WT and CRISPR-deleted embryos at e16.5, cartilage in blue, bone in red (Adapted from [23] with Open Access).

Earlier investigations of structural and copy number variants were performed by functional genomics using transgenic reporter assays and laborious generation of knock-in/knockout mice via cis/trans recombination of *loxP* sites and mouse-crossing steps, a process that can take up to 12 months. However, newer genomic techniques have been developed to generate these variants more efficiently in mice for the ready availability of mimics for structural and copy-number variants implicated in human genetic diseases. Kraft and colleagues in the Mundlos group investigated the use of CRISPR/Cas to generate structural variants, including inversions, and copy-number gains and losses [23]. CRISPR/Cas is a rapid technique, yielding results in the order of 10 weeks, and uses two flanking synthetic guide RNAs (sgRNAs) targeted to distinct positions on a chromosome to cause inversions, deletions, and duplications within the intervening region via non-homologous end joining (NHEJ) (Figure 3). The group was able to generate multiple CRISPR/Cas constructs ranging in size from ~1 kb to 1.6 Mb in embryonic stem cells (ESCs) and disrupted multiple mouse genes, including *Pitx1*, *Laf4*, and *Epha4*. Disruption of *Laf4* in the CRISPR-Cas mouse model recapitulated the structural abnormalities observed in a human patient with Nievergelt syndrome, with similar disruption in the syntenic *LAF4* region and presentation of lower-limb abnormalities, including a small triangular ossification center of the tibia and severe hypoplasia of the fibula (Figure 4).

Further work from the Mundlos group by Lupiáñez and colleagues confirmed via Hi-C data that human and mouse cells shared a highly similar TAD structure and that mice could serve as an in vivo model system for genetic diseases involving structural and copy-number variants [24]. The group used CRISPR/Cas to generate structural variants within a TAD encompassing the mouse gene *Epha4*, and flanked by TADs containing *Wnt6*, *Ihh*, and *Pax3,* which is syntenic with the human TADs containing *WNT6*/*IHH*/*EPHA4*/*PAX3*. Multiple CRISPR/Cas constructs were created in this genomic region to recapitulate the human genetic diseases brachydactyly, F-syndrome, and polydactyly, with disruption of TAD boundaries leading to ectopic interactions between *Pax3*, *Wnt6*, and *Ihh*, respectively, with the enhancers within the *Epha4* TAD. Similar results were seen in human embryonic stem cells from patients with the aforementioned diseases. To determine the native sites of action of the enhancers within the *Epha4* TAD, they used e11.5 mice to screen for *Epha4* enhancers selected from ChIP-Seq data that had activity in limb buds with a *lacZ* reporter assay. This work was shown to provide a framework for the interpretation of structural variants in human genetic disease through the examination of TADs and TAD boundaries. Similar work by Rajderkar and colleagues in the Pennacchio group confirmed the necessity of intact TAD boundaries for normal genome function via intensive targeted deletion of CTCF-binding regions at these boundaries [25].

In addition to functional genomics research in embryonic mice, some groups utilized zebrafish (*Danio rerio*) to investigate the function of enhancer sequences in an alternative vertebrate model. Zebrafish diverged from the mammalian lineage ~420 million years ago, have ~70% conservation with human protein-coding genes, and are easy to genetically manipulate and propagate [26,27]. Work by Booker and colleagues examined 22 human sequences that also functioned as mouse limb enhancers, including the ZRS element that regulates *SHH* gene expression during limb development, to test their activity in a zebrafish model [28]. They found that 10/22 enhancer sequences were positive for pectoral fin activity, with the ZRS showing expression in the zebrafish forebrain only. Notably, these ten sequences had activity in other tissues as well, and five of these ten fin enhancers were not conserved in fish. They posited that mammalian enhancers have various activity domains in zebrafish and that sequence conservation may not be an ideal filter to identify functionally conserved regulatory elements. Although some enhancers may function differently in zebrafish models, the overall retention of TAD synteny between humans, mice and zebrafish enables zebrafish models of structural variation in disease to remain useful [29,30].

As earlier techniques in functional genomics like ChIP-Seq, Hi-C, and CRISPR/Cas mature, newer tools are emerging to further investigate the 3D genome. A newer technique called CUT&RUN (Cleavage Under Targets & Release Using Nuclease) has been developed to interrogate protein/DNA interactions and overcome some of the difficulties of ChIP, including systematic biases and artifacts inherent in the method, by using DNA fragmentation and solubilization of total chromatin [31]. CUT&RUN results in lower background because the method is performed on intact nuclei and without whole-genome fragmentation, which leads to background noise and the need for normalization. Preliminary use in yeast and human cells for mapping chromatin-associated complexes is promising. Early work by Liu and colleagues used CUT&RUN in human erythroid HUDEP-2 cells to determine the binding motif for BCL11A protein to modulate expression of γ-globin in hereditary persistence of fetal hemoglobin (HPFH) syndrome [32]. Prior research using ChIP-Seq to determine the recognition sequence of BCL11A in the regulation of expression of γ-globin was inconclusive. Because of CUT&RUN’s ability to use fewer cells and to detect smaller DNA fragments (<40 bp), the group found the motif ‘TGACCA’ as the preferred in vivo binding sequence of BCL11A and that BCL11A bound the distal γ-globin promoter most strongly for repression. More recent work by Holt and colleagues used CUT&RUN in a mouse model of human stress response [33]. Their work showed that the H3K27me1 histone modification is a marker of early-life stress through increased neuronal excitability and serves as a “chromatin scar” that modulates neuronal plasticity to mediate lasting susceptibility to stress. As this new technique comes to wider acceptance, CUT&RUN may eventually replace ChIP for transcription factor profiling.

These aforementioned functional genomics techniques have the ability to characterize the genetic and genomic content of potentially pathogenic SVs and CNVs in intergenic regions via the determination of enhancers and other regulators of gene expression, and possible disruption of TADs. This could lead to definitive diagnoses in patients where a causative sequence variant, CNV or SV was not determined by prior genomic testing.

## 3. Recent Case Studies Characterizing SVs in Humans

Historical cases of genomic abnormalities that have been well-characterized by functional genomics include what are called enhanceropathies [6], or pathogenic alterations of enhancer function, such as preaxial polydactyly (PPD; Involving regulation of the *SHH* gene) [7], Pierre Robin syndrome (*SOX9*) [8], and 5q14.3 microdeletion syndrome (*MEF2C*) [9]. Functional assays determined that sequences within intron 5 of the human *LMBR1* gene, which were ~800 kb away from the Sonic Hedgehog (*SHH*) gene on 7q36.3, were responsible for proper expression of SHH protein in limb development. Translocations or other structural variations that disrupted the intron 5 region of *LMBR1* resulted in the PDD phenotype [7]. Pierre Robin syndrome (PRS), presenting with micrognathia, glossoptosis, and cleft palate, was observed in multiple families with reciprocal translocations of 17q24, flanking both sides of the *SOX9* gene region at ~1.5 Mb distance. Disruptions in either of these regions that perturbed regulatory elements resulted in PRS. Microdeletions or translocations in 5q14.3 that disrupt the expression of *MEF2C* result in severe ID, epilepsy and/or cerebral malformations. Inversions or deletions flanking *MEF2C* were shown to disrupt the TAD harboring *MEF2C* and uncouple the expression of the MEF2C protein [9,34].

Axenfeld–Rieger syndrome (ARS) is a rare autosomal dominant genetic disease with primarily ocular involvement and glaucoma. Mitchell and colleagues described two patient families with ARS and the use of functional genomics to determine the cause [35]. ARS is typically caused by pathogenic coding variants in the genes *PITX2* or *FOXC1*. *PITX2* encodes a homeobox transcription factor active in the developing eye during embryogenesis. However, no apparent abnormalities in *PITX2* or *FOXC1* coding sequences were detected. Unique to these two families were disruptions in the adjacent enhancer locus of *PITX2*. After initial chromosomal microarray, exome and genome sequencing, one family was found to have a 450 kb deletion of the enhancer region, and the other family had a large 12.54 Mb inversion that uncoupled *PITX2* from the enhancer locus. Both of these abnormalities, a copy number variant and a structural variant, were predicted to uncouple *PITX2* from its enhancer machinery and thus lead to underexpression similar to a true *PITX2* deletion or pathogenic sequence variant.

Heterotopic ossification (HO) is a genetic disorder caused by abnormal mineralization of soft tissues, with putative involvement by the BMP, TGFβ and WNT signaling pathways and the *ACVR1* gene. Melo and colleagues described a female patient with HO that progressed rapidly with ossification and early demise [36]. After extensive testing with array-CGH, whole-genome and Sanger sequencing, a duplication of 820 kb of genomic material from 2p13 was shown to be inserted into Xq26.1, resulting in the disruption of a TAD and the placement of enhancer regions of 2p13 ectopically adjacent to *ARHGAP36* and subsequent enhancer hijacking. This caused inappropriate overexpression of ARHGAP36 protein in the soft tissues of this patient, induction of early osteogenic differentiation markers, extracellular matrix proteins, and ossification.

## 4. Limitations

While cytogenomic techniques like karyotyping, FISH, and microarray have advantages and disadvantages regarding resolution, sensitivity, accessibility and cost [37], functional genomics techniques, as complementary methods to cytogenomics, have their own advantages and disadvantages (Table 1). Hi-C provides genome-wide coverage and confers 3D spatial information of chromatin interactions, but has limited resolution [34]. ChIP-Seq provides massively parallel functional annotation of regulatory states but requires specific, high-quality antibodies and samples [38]. CUT&RUN has reduced sample input requirements and higher sensitivity relative to ChIP-Seq, but has yet to be standardized for clinical usage [15,33]. Long-read sequencing, not a functional assay per se, has the ability to span long genomic repeats and detect balanced structural rearrangements like translocations and inversions, phasing of haplotypes, and precise mapping of genomic breakpoints, which can integrate with cytogenomic data and 3D mapping to provide a more complete description of potentially pathogenic SVs and CNVs. However, long-read sequencing has higher read error rates and incurs a higher cost to store larger sequencing datasets [15].

## 5. Future Directions

As newer cytogenomic diagnostic tests like optical genome mapping (OGM) and genomic proximity mapping (GPM) detect increasing numbers of cryptic or complex structural variants [37], this will lead to additional functional cytogenomics studies with newer functional genomics tools like CUT&RUN. Assays currently in development include spatial transcriptomics and transcriptome-wide association studies (TWAS) [39], along with single-cell CUT&RUN [40,41] and other single-cell assays [42]. Spatial transcriptomics can provide spatio-transcriptional architecture to tumors in multiple individuals, for example, in triple-negative breast cancer (TNBC), despite intratumoral and stromal heterogeneity within individual samples. Eventually, as the technology matures, the future possibilities for CRISPR/Cas-based therapies to treat genetic and genomic disease, including enhanceropathies, look promising [43].

## 6. Discussion/Summary

Functional genomics has expanded over the last few decades from the discovery of ultraconserved genomic sequences between humans, mice, other mammals and even fish, to the development of powerful tools to test expression of these sequences in reporter assays in embryonic mice and zebrafish. Leveraging this information has enabled the use of powerful methods such as CRISPR/Cas for genome editing in mouse or human cells to interrogate human and other mammalian genomes for insights into the structure and regulation of the genome in three dimensions within the nucleus. This has enabled more accurate diagnosis of human genetic and genomic disease by examining the potential pathogenic effects of SVs and CNVs on gene regulation and expression. As clinical genomics advances in detecting structural and copy number variants, functional cytogenomics will advance as well to provide answers.

## Figures and Tables

**Table 1:** Advantages and disadvantages of functional genomics assays presented (Adapted from [15] with Open Access).

Technique	Principle	Strengths	Limitations
Hi-C	Genome-wide sequencing of chromatin interactions including altered TADs, chromatin loops, and structural rearrangements in constitutional and neoplastic genetic disease	Genome-wide coverage, 3D spatial information	Limited resolution without enrichment
ChIP-Seq	Sequencing of DNA bound to histone marks or TFs via immunoprecipitation, providing epigenetic profiling in developmental disorders and cancer	Functional annotation of regulatory states	Requires specific antibody and good-quality samples
Long-Read Sequencing	Sequencing of reads >10~100 kb in length	Allows spanning of repeat sequences, phasing, and precise breakpoint detection	Higher error rates than short-read sequencing, cost of data storage
CUT&RUN	Epigenetic profiling with minimal material input, eventually rapid profiling of clinical specimens	Low input requirements, high sensitivity	Not yet standardized for clinical use

## Data Availability

No new data were created or analyzed in this study.
